# Goodman and Kruskal’s Gamma Coefficient for Ordinalized Bivariate Normal Distributions

**DOI:** 10.1007/s11336-020-09730-5

**Published:** 2020-10-27

**Authors:** Alessandro Barbiero, Asmerilda Hitaj

**Affiliations:** 1 Department of Economics, Management, and Quantitative Methods, Università degli Studi di Milano, Via Conservatorio, 7, 20122 Milan, Italy; 2 Department of Economics, Università degli Studi dell’Insubria, via Monte Generoso 71, 21100 Varese, Italy

**Keywords:** Bivariate normal distribution, Discretization, Gamma coefficient, Latent variable, Ordinal association

## Abstract

We consider a bivariate normal distribution with linear correlation $\rho$ whose random components are discretized according to two assigned sets of thresholds. On the resulting bivariate ordinal random variable, one can compute Goodman and Kruskal’s gamma coefficient, $\gamma$, which is a common measure of ordinal association. Given the known analytical monotonic relationship between Pearson’s $\rho$ and Kendall’s rank correlation $\tau$ for the bivariate normal distribution, and since in the continuous case, Kendall’s $\tau$ coincides with Goodman and Kruskal’s $\gamma$, the change of this association measure before and after discretization is worth studying. We consider several experimental settings obtained by varying the two sets of thresholds, or, equivalently, the marginal distributions of the final ordinal variables. This study, confirming previous findings, shows how the gamma coefficient is always larger in absolute value than Kendall’s rank correlation; this discrepancy lessens when the number of categories increases or, given the same number of categories, when using equally probable categories. Based on these results, a proposal is suggested to build a bivariate ordinal variable with assigned margins and Goodman and Kruskal’s $\gamma$ by ordinalizing a bivariate normal distribution. Illustrative examples employing artificial and real data are provided.

## Introduction

The use of the multivariate normal distribution as a latent construct for modelling observed correlated or associated discrete or ordinal variables can be dated back to the seminal book by Lazarsfeld and Henry (1968) and to the later work by Muthen (1983), in the context of structural equation modeling. Latent variable modelling has since then gradually become an integral part of mainstream statistics and is currently used for a multitude of applications in different subject areas (Beaujean 2014).

It is also an indisputable fact that the ability to simulate artificial data resembling the main features of some observed dataset or following the specifications of a study design is necessary when comparing and investigating the behaviour of statistical procedures and exploring their robustness; such features or specifications can often be conveniently summarized by the empirical marginal distributions and pairwise measures of correlation or association.

In this work, we will focus our attention on the relationship between a concordance measure of the bivariate normal latent variable (Kendall’s rank correlation, directly related to the more popular Pearson’s correlation) and a corresponding association measure of the ordinalized variable (Goodman and Kruskal’s gamma). On the one hand, we will analyze how the correlation of the latent variable and the marginal distributions of the final ordinal variables affect their correlation/association: we can call it the “direct problem”. Specifically, we will investigate the effect of the number of categories and the probability distribution (uniform/symmetrical/skewed) on the distortion of the association measure. On the other hand, we will provide a procedure that allows construction and simulation of a random vector of discrete variables with assigned marginal distributions and association by discretizing a bivariate normal random vector with a pairwise correlation that is able to induce the desired association among the ordinal variables (the “inverse problem”, i.e., finding the parameters of the continuous model that produce the (parameters of the) observed ordinal variables).

The paper is structured as follows. In the next section, we will recall the definition of Goodman and Kruskal’s gamma coefficient and summarize its main properties, also in comparison with alternative measures of ordinal association. In Sect. 3, we will analyze the change in magnitude of association before and after ordinalization of a bivariate normal variable, measured by Kendall’s tau and Goodman and Kruskal’s gamma, respectively, under a wide array of experimental conditions. Section 4 suggests and examines a procedure for building a bivariate ordinal variable with assigned marginal distributions and association. Section 5 proposes a possible application to inference of the algorithm of Sect. 4; Sect. 6 presents an illustrative example with real data. The last section is devoted to some final remarks and possible research prospects.

## Measures of Ordinal Association: Goodman and Kruskal’s Gamma

Measures of ordinal association between two variables use the property that the categories of ordinal variables have a natural order; but the way in which these measures use this information may differ considerably. Some measures are based on the intuitive notion that ordinal association should have an interpretation analogous to that of association for metric variables, say, Pearson’s correlation; in this group, we find Spearman’s $\rho$, Kendall’s $\tau$, polychoric correlation, Somer’s *d*, and Goodman-Kruskal’s $\gamma$. These coefficients allow us to make statements of the general form “if scores on *X* increase, then most probably, scores on *Y* will increase” (Kampen and Swyngedouw 2000). Other measures of ordinal association have a background in information theory and have interpretations in terms of stochastic entropy (Bryson and Phillips 1975; Laird 1979; Gilula et al. 1988). Henceforth, we will focus on the former family of measures.

Let us consider a pair of ordinal variables (*X*, *Y*), with *H* and *K* ordered categories, respectively, and introduce the concept of concordance/discordance by considering two independent realizations $(x_i,y_i)$ and $(x_j,y_j)$. $(x_i,y_i)$ and $(x_j,y_j)$ will be said to be concordant if $x_i<x_j$ and $y_i<y_j$, or if $x_i>x_j$ and $y_i>y_j$. Conversely, $(x_i,y_i)$ and $(x_j,y_j)$ will be said to be discordant if $x_i<x_j$ and $y_i>y_j$, or if $x_i>x_j$ and $y_i<y_j$. Thus, one can define the probability of concordance as

$$\begin{aligned} \varPi _c = Pr\left\{ X_i<X_j \text { and } Y_i<Y_j \right\} + Pr\left\{ X_i>X_j \text { and } Y_i>Y_j \right\} \end{aligned}$$

and similarly the probability of discordance as

$$\begin{aligned} \varPi _d = Pr\left\{ X_i<X_j \text { and } Y_i>Y_j \right\} + Pr\left\{ X_i>X_j \text { and } Y_i<Y_j \right\} . \end{aligned}$$

The gamma coefficient belongs to a larger family of ordinal correlation measures (see, e.g., Woods 2009, for an exhaustive account); it is defined as the following ratio (see, e.g., Agresti 2010, pp.186–187):

1 $$\begin{aligned} \gamma =\frac{\varPi _c - \varPi _d}{\varPi _c+\varPi _d} \end{aligned}$$

$\varPi _c$ and $\varPi _d$ can be conveniently expressed in terms of the joint probabilities $p_{ij}=P(X=x_i,Y=y_j)$:

$$\begin{aligned} \varPi _c=2\mathop {\sum \sum }_{i<h}\mathop {\sum \sum }_{j<k} p_{ij}p_{hk},\quad \varPi _d=2\mathop {\sum \sum }_{i<h}\mathop {\sum \sum }_{j>k} p_{ij}p_{hk}. \end{aligned}$$

By defining the quantities

$$\begin{aligned} p_{hk}^{(c)} = \sum _{a<h}\sum _{b<k} p_{ab} + \sum _{a>h}\sum _{b>k} p_{ab} \end{aligned}$$

and

$$\begin{aligned} p_{hk}^{(d)} = \sum _{a<h}\sum _{b>k} p_{ab} + \sum _{a>h}\sum _{b<k} p_{ab} \end{aligned}$$

for each $h=1,\dots ,H$, $k=1,\dots ,K$, then $\varPi _c$ and $\varPi _d$ can be rewritten as

$$\begin{aligned} \Pi _c =\sum _{h=1}^H\sum _{k=1}^K p_{hk}p_{hk}^{(c)} \text {, } \Pi _d =\sum _{h=1}^H\sum _{k=1}^K p_{hk}p_{hk}^{(d)}. \end{aligned}$$

For a bivariate continuous random variable, $\gamma$ can be still computed through Eq. (1): in this case, since the probability of concordance and the probability of discordance sum up to 1 (there is no probability of tied values for either *X* or *Y*), then $\gamma =\varPi _c-\varPi _d=2\varPi _c-1$, and it coincides with Kendall’s rank correlation $\tau$ (Kendall 1945), simply defined as the difference between the probability of concordance and the probability of discordance. We recall that for two continuous random variables *X* and *Y*, Kendall’s $\tau$ depends only on their unique copula *C* (representing the dependence structure of the bivariate random vector (*X*, *Y*); see McNeil et al. , 2005, chapter 5), specifically,

$$\begin{aligned} \tau (X,Y) = 4\int _0^1\int _0^1 C(u_1, u_2) \text {d}C(u_1, u_2) - 1, \end{aligned}$$

and not on the marginal distributions of *X* and *Y*. $\tau$ ranges between $-1$ and $+1$; it attains the upper bound if and only if *X* and *Y* are comonotonic, whereas it attains the lower bound if and only if *X* and *Y* are countermonotonic.

Like Pearson’s correlation and other correlation measures such as Spearman’s rho and the aforementioned Kendall’s tau, $\gamma$ takes values in $[-1,+1]$. The values $-1$, 0, and $+1$ are attained when $\varPi _c=0$, $\varPi _c=\varPi _d$, $\varPi _d=0$, respectively. There are however some potential problems with the gamma coefficient, which were immediately recognized by Goodman and Kruskal (1954):

- $\gamma$ is unstable over various “cutting points”, that is to say, $\gamma$ tends to increase as the categories of a contingency table are collapsed, since $\gamma$ gives no consideration to tied pairs, as can be seen from Eq. (1), and the number of tied pairs increases as the table is collapsed;
- $\gamma$ also usually yields greater association values than other measures of ordinal association, as it does not consider any of the tied pairs;
- Finally, $\gamma$ is a weakly monotonic measure of ordinal association, i.e., it reaches $+1$ under a variety of cell frequency configurations, not only in case of strict perfect association (Berry et al. 2018). For example, the bivariate distribution of Table 1, where all the joint probabilities are zero except those labelled with $\times$, presents a value of $\gamma$ equal to $+1$, although the relationship between *X* and *Y* is not perfectly monotonic (Kendall’s $\tau$ and Spearman’s $\rho$ would be strictly smaller than 1).

With the objective of overcoming these pitfalls, some modifications to the gamma coefficient or alternative measures of ordinal association have been proposed (Rousson 2007; Kvålseth 2017, 2018).

**Table 1:** Graphic for a weakly monotonic relationship

	1	2	3	4	5	6
1	$\times$	$\times$				
2		$\times$	$\times$			
3			$\times$	$\times$		
4				$\times$	$\times$	
5					$\times$	$\times$
6						$\times$

A remark on the possible extension of the gamma coefficient to higher dimensions can be made. Let us consider the *d*-variate ordinal random vector $\pmb {X}=(X_1, X_2,\dots , X_d)^\intercal$, with $d>2$; then, a $\gamma$ association matrix remains defined as

$$\begin{aligned} \Gamma (\pmb {X})=[\gamma _{ij}]_{i=1,\dots ,d;j=1,\dots ,d} \end{aligned}$$

with $\gamma _{ij}=\gamma (X_i,X_j)$. This matrix however is not necessarily positive semidefinite, as happens for Spearman’s or Kendall’s rank correlation matrices, which are both valid correlation matrices (see McNeil et al. 2005, p.207). An easy counterexample is provided as follows. Consider the trivariate distribution of Table 2. The values of $\gamma$ for the bivariate distributions (*X*, *Y*), (*X*, *Z*), and (*Y*, *Z*) are $\gamma _{xy}=1/3$, $\gamma _{xz}=1$, and $\gamma _{yz}=-7/9$, respectively. The corresponding $\gamma$ association matrix is:

$$\begin{aligned} \Gamma (X,Y,Z)=\begin{bmatrix} 1 &{} 1/3 &{} 1\\ 1/3 &{} 1 &{} -7/9 \\ 1 &{} -7/9 &{} 1 \end{bmatrix}; \end{aligned}$$

its three eigenvalues are 2.119, 1.322, and $-0.441$; since one of them is negative, the matrix is clearly not positive semidefinite.

**Table 2:**
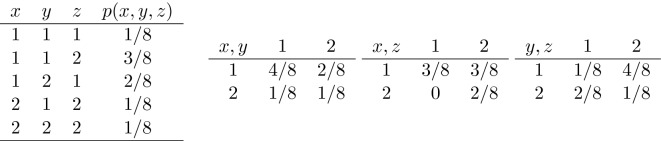
A trivariate probability distribution and its corresponding bivariate marginal distributions

With *n* pairs of observations constituting a multinomial sample, the sample analog of $\gamma$ is given by ${\hat{\gamma }} = (C-D)/(C+D)$, with *C* the number of concordant pairs and *D* the number of discordant pairs. It is possible to prove that $\sqrt{n}({\hat{\gamma }}-\gamma )$ is asymptotically normal with mean zero and variance given by

$$\begin{aligned} {\sigma }^2 = \sum _i\sum _j p_{ij} \phi _{ij}^2/(\Pi _c+\Pi _d)^4 = \frac{16}{(\Pi _c+\Pi _d)^4}\sum _i\sum _j p_{ij}\left( \Pi _cp_{ij}^{(d)} - \Pi _dp_{ij}^{(d)}\right) ^2 \end{aligned}$$

where $\phi _{ij}=4(\Pi _d p_{ij}^{(c)} - \Pi _c p_{ij}^{(d)})$ (see, e.g., Agresti 2010).

In practice, replacing $p_{ij}$, $\Pi _c$, and $\Pi _d$ with their sample values in ${\sigma }^2$ yields the ML estimate ${\hat{\sigma }}^2$ of $\sigma ^2$. The term $SE = {\hat{\sigma }}/\sqrt{n}$ is an estimated standard error for ${\hat{\gamma }}$, and Wald confidence interval for $\gamma$ is $({\hat{\gamma }} \pm z_{1-\alpha /2}SE)$.

Rosenthal (1966); Gans and Robertson (1981) showed that ${\hat{\gamma }}$ has a tendency to converge slowly to normality and to have distributional irregularity, bias, and skewness problems, especially when the true absolute value of $\gamma$ is large. O’Gorman and Woolson (1988); Carr et al. (1989) pointed out that better convergence occurs using the Fisher-type transform ${\hat{\xi }} = \frac{1}{2}\log [(1+{\hat{\gamma }})/(1-{\hat{\gamma }})]$, whose asymptotic variance equals the asymptotic variance of ${\hat{\gamma }}$ multiplied by $(1 - \gamma ^2)^{-2}$. A confidence interval can be constructed for $\xi$ and then inverted to one for $\gamma$, by using the inverse transformation ${\hat{\gamma }} = (e^{2{\hat{\xi }}}-1)/(e^{2{\hat{\xi }}}+1)$ .

## Analysis of the Relationship Between $\rho$ and $\gamma$ for Ordinalized Bivariate Normal Distribution

Discretization of continuous variables is commonly encountered in practice. Based on observed nominal age, income, temperature, and depression score, one can derive ordinal variables such as young-middle-old age, low-medium-high income, cold-cool-average-hot temperature, and no-mild-moderate-severe depression. Discretization is usually avoided by statisticians for intuitive and valid reasons, the most prominent of which is the associated power and information loss. Some problems related to identification of statistical models obtained by discretization have been recently raised by Grønneberg and Foldnes (2019). However, simplicity, better interpretability and comprehension of the effects of interest, and superiority of some categorical data measures such as odds ratio have been argued by proponents of discretization (Liu et al. 2002).

The objective of this section is the determination of association magnitude changes when the univariate components of a bivariate normal distribution are ordinalized, i.e., the range of each continuous component is divided into contiguous intervals, which are assigned an ordered category or a consecutive positive integer. A similar and extensive analysis has been conducted by Demirtas and Vardar-Acar (2017) to investigate the effects of discretization of continuous variables on the magnitude of linear correlation. If the underlying continuous distribution is bivariate normal, it can be proved that discretization always preserves the sign of linear correlation and more importantly leads to a reduction in magnitude. This result, which has been empirically observed in many simulation studies (see, e.g., Bollen and Barb 1981) and was claimed to hold only “in large samples” by Demirtas and Vardar-Acar (2017), is just a consequence of a previous, more general theoretical result, named Lancaster’s theorem (Lancaster 1957), which states that the correlation of a bivariate normal cannot increase whatever transformations are applied to its univariate components (see also Mari and Kotz 2001, p.155, where it is reported as an “extremal property” of the bivariate normal distribution).

For a bivariate normal distribution with correlation coefficient $\rho$, the following relationship holds between $\rho$ and Kendall’s rank correlation $\tau$:

2 $$\begin{aligned} \tau =\frac{2}{\pi } \arcsin \rho . \end{aligned}$$

Equation (2) holds also for most elliptical distributions, e.g., for bivariate Student’s *t*, and for most bivariate distributions whose dependence structure is described by an elliptical copula (McNeil et al. 2005). If both univariate margins are discretized/ordinalized through pre-specified thresholds, one can compute some measure of ordinal association based on the resulting bivariate ordinal variable, such as Goodman and Kruskal’s $\gamma$: we know (see the previous section) that for continuous bivariate random variables, such as the bivariate normal, the definitions of $\gamma$ and $\tau$ coincide. Thus, it makes sense to analyze the relationship between $\tau$ and $\gamma$ as a measure of the change in association before and after ordinalization.

Let us start from a very simple case, i.e., dichotomization of the margins of a standard bivariate normal random variable $(Z_1,Z_2)$, with zero marginal means, unit marginal variances, and linear correlation $\rho$. Consider the bivariate ordinal variable (*X*, *Y*) obtained as follows:

3 $$\begin{aligned} X={\left\{ \begin{array}{ll} x_1 &{}\quad Z_1\le 0\\ x_2 &{}\quad Z_1>0 \end{array}\right. },\quad Y={\left\{ \begin{array}{ll} y_1 &{} \quad Z_2\le 0\\ y_2 &{} \quad Z_2>0 \end{array}\right. }, \end{aligned}$$

with $x_1<x_2$ and $y_1<y_2$ being two ordered categories. Therefore, the marginal probabilities for *X* and *Y* are $P(X=x_1)=P(X=x_2)=1/2$ and analogously $P(Y=y_1)=P(Y=y_2)=1/2$. Since we know that for a bivariate normal distribution $P(Z_1\le 0,Z_2\le 0)=P(Z_1> 0,Z_2> 0)=\frac{1}{4} + \frac{1}{2\pi }\arcsin \rho$ (see, e.g., McNeil et al. 2005), then the joint probability of (*X*, *Y*) is that displayed in the following table:

$$\begin{aligned} \begin{array}{cccc} X,Y &{} y_1 &{} y_2 &{} total\\ \hline x_1 &{} 1/4+1/(2\pi )\arcsin \rho &{}\quad \quad 1/4-1/(2\pi )\arcsin \rho &{} 1/2 \\ x_2 &{} 1/4-1/(2\pi )\arcsin \rho &{}\quad \quad 1/4+1/(2\pi )\arcsin \rho &{} 1/2\\ \hline total &{} 1/2 &{} 1/2 &{} 1\\ \end{array} \end{aligned}$$

**Fig. 1:**
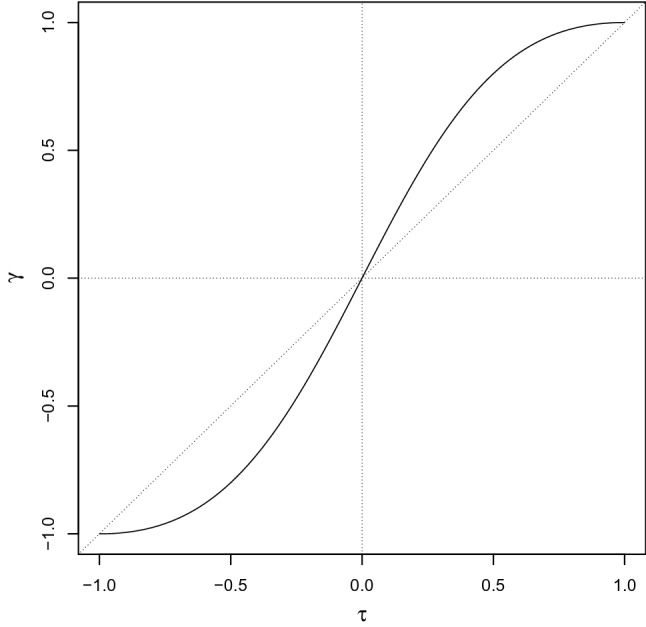
Graph of the gamma coefficient for a dichotomized bivariate normal random variable with Kendall’s rank correlation $\tau$, see Eq. (5). The dashed line is the 45 degrees line passing through the origin

Based on it, by using Eq. (1), one can compute the gamma coefficient as a function of $\rho$:

4 $$\begin{aligned} \gamma = \frac{4\pi \arcsin \rho }{\pi ^2+ 4(\arcsin \rho )^2}, \end{aligned}$$

or, recalling (2), in terms of $\tau$:

5 $$\begin{aligned} \gamma = \frac{2\tau }{1+\tau ^2}, \end{aligned}$$

whose graph is plotted in Fig. 1. This graph clearly shows how Goodman-Kruskal’s $\gamma$ for the dichotomized random variable is a strictly increasing and odd function of its analogue Kendall’s $\tau$ for the bivariate normal distribution and, in absolute value, is always larger than or equal to $\tau$, the equality holding when $\tau =0$ (i.e., when the two normal components are independent), when $\tau$ equals 1 (perfectly positively correlated normal random variables), or when $\tau$ equals $-1$ (perfectly negatively correlated normal random variables). It is worth noting that in this context, $\gamma$ is equal to $+1$ ($-1$) only in case of a perfect monotonic (countermonotonic) relationship between *X* and *Y*.

By choosing thresholds different from zero for the random components $Z_1$ and $Z_2$, we can numerically obtain the value of $\gamma$ corresponding to any value of $\tau$. Similarly, one can discretize either or both continuous random components into more than two categories, applying different sets of thresholds (or, equivalently, assigning different marginal distributions to the final ordinal variables). In general, let us consider a bivariate standard normal distribution $(Z_1,Z_2)$ whose two continuous components are discretized into two ordinal variables *X* and *Y* according to the following scheme:

$$\begin{aligned} X={\left\{ \begin{array}{ll} x_1 &{} Z_1\le \theta _1\\ x_2 &{} \theta _1< Z_2\le \theta _2\\ \cdots \\ x_H &{} \theta _{H-1}< Z_1\le \theta _H=+\infty \end{array}\right. }, \quad Y={\left\{ \begin{array}{ll} y_1 &{}\quad Z_2\le \eta _1\\ y_2 &{}\quad \eta _1<Z_2\le \eta _2 \cdots \\ y_K &{}\quad \eta _{K-1}< Z_2\le \eta _K=+\infty \end{array}\right. } \end{aligned}$$

where $\theta _1<\theta _2<\dots <\theta _H=\infty$ and $\eta _1<\eta _2<\dots <\eta _K=\infty$ constitute two sets of thresholds. If we denote with *F*(*i*, *j*) the bivariate joint cumulative probability $P(X\le x_i,Y\le y_j)$ of the bivariate ordinal variable (*X*, *Y*), $i=1,\dots ,H$, $j=1,\dots ,K$, and let $F_1(i):=P(X\le x_i)$ and $F_2(j):=P(Y\le y_j)$ be the two marginal cumulative distributions, we have $F_1(i)=\Phi (\theta _i)$ and $F_2(j)=\Phi (\eta _j)$, and the joint cumulative distribution function (cdf) of (*X*, *Y*) is

6 $$\begin{aligned} F(i,j)=\Phi _{\tau }(\theta _i,\eta _j)=\Phi _{\tau }(F_1^{-1}(i),F_2^{-1}(j)), \end{aligned}$$

where $\Phi _{\tau }$ is the joint cdf of a bivariate normal with standard components and rank correlation $\tau$, and $F_1^{-1}$ ($F_2^{-1}$) is the generalized inverse of $F_1$ ($F_2$). The joint probabilities $p_{ij}=P(X=x_i,Y=y_j)$ are then obtained as

7 $$\begin{aligned} p_{ij} = F(i,j) - F(i-1,j) - F(i,j-1) + F(i-1,j-1) = \sum _{t=0}^1\sum _{v=0}^1 F(i-t,j-v)\cdot (-1)^{t+v}\nonumber \\ \end{aligned}$$

and based on these $p_{ij}$, the gamma coefficient can be computed using Eq. (1).

In the following study, we will consider three different possible types of ordinal marginal distributions, namely, (i) uniform (i.e., equiprobable categories) (ii) symmetrical (non-uniform), based on normal scores, and (iii) asymmetrical (triangular). We will examine what kind of relationship $\tau$ and $\gamma$ have under these three macro-settings by considering the same distribution for the two margins and varying the number of categories. Relevant code developed in the R environment (R Core Team 2020) is freely available as supplementary material.

**Fig. 2:**
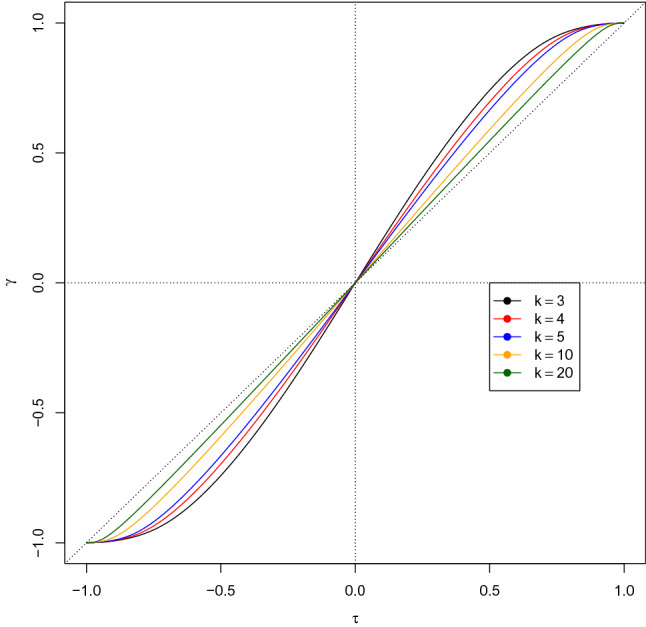
Kruskal’s $\gamma$ for an ordinalized bivariate normal random variable with Kendall’s rank correlation $\tau$; the categories of the two ordinal variables are set both equal to an integer *K* and have uniform probabilities 1/*K*. The dashed line is the 45 degrees line passing through the origin

Figure 2 displays the graphs of the $(\tau ,\gamma )$ curve when the two marginal distributions have the same number of equiprobable categories $H=K=3,4,5,10,20$. Table 3 reports the values of $\gamma$ for $\tau$ ranging from $-1$ to $+1$, with step length of 0.1, and several values of the common number of categories of the two identical margins ($H=K=2, 3, 5, 7, 10, 20, 50, 100$). Note that the $(\tau ,\gamma )$ curve, for a given *H*, is strictly increasing and always passes through the points $(-1,-1)$, (0, 0), and (1, 1), and, as one can expect, by increasing *H* (i.e., when the ordinal distributions “resemble” a continuous one), it tends to get close to the 45-degree line passing through the origin. Note that $\gamma$ is an odd function of $\tau$, i.e., $\gamma (\tau )=-\gamma (-\tau )$. For a given value of $\tau >0$, $\gamma$ is a decreasing function of *H*; for a given value of $\tau <0$, $\gamma$ is an increasing function of *K*; as *K* tends toward $\infty$, $\gamma$ seems to converge, though slowly, to $\tau$. It is worth observing, however, that even for a moderately large number of categories, there is a substantial difference between the values of $\tau$ and $\gamma$ before and after ordinalization. When $H=100$, there is still a relative difference of up to $2\%$ between the values of $\tau$ and $\gamma$.

**Table 3:** Values of Goodman and Kruskal’s gamma for several combinations of $\tau$ and number of categories of the two identical marginal triangular distributions

$\tau ,K$	2	3	5	7	10	20	50	100
$-$1	$-$1.00000	$-$1.00000	$-$1.00000	$-$1.00000	$-$1.00000	$-$1.00000	$-$1.00000	$-$1.00000
$-$0.9	$-$0.99448	$-$0.99321	$-$0.99036	$-$0.98698	$-$0.98016	$-$0.95799	$-$0.92968	$-$0.91622
$-$0.8	$-$0.97561	$-$0.97031	$-$0.95286	$-$0.93261	$-$0.90805	$-$0.86492	$-$0.82918	$-$0.81522
$-$0.7	$-$0.93960	$-$0.92266	$-$0.87790	$-$0.84384	$-$0.81053	$-$0.76170	$-$0.72647	$-$0.71358
$-$0.6	$-$0.88235	$-$0.84482	$-$0.77885	$-$0.73856	$-$0.70299	$-$0.65525	$-$0.62312	$-$0.61176
$-$0.5	$-$0.80000	$-$0.74150	$-$0.66519	$-$0.62433	$-$0.59043	$-$0.54731	$-$0.51950	$-$0.50986
$-$0.4	$-$0.68966	$-$0.61803	$-$0.54185	$-$0.50458	$-$0.47493	$-$0.43854	$-$0.41572	$-$0.40792
$-$0.3	$-$0.55046	$-$0.47841	$-$0.41176	$-$0.38119	$-$0.35756	$-$0.32926	$-$0.31185	$-$0.30596
$-$0.2	$-$0.38462	$-$0.32625	$-$0.27699	$-$0.25537	$-$0.23898	$-$0.21966	$-$0.20792	$-$0.20398
$-$0.1	$-$0.19802	$-$0.16537	$-$0.13923	$-$0.12805	$-$0.11966	$-$0.10987	$-$0.10397	$-$0.10199
0	0.00000	0.00000	0.00000	0.00000	0.00000	0.00000	0.00000	0.00000
0.1	0.19802	0.16537	0.13923	0.12805	0.11966	0.10987	0.10397	0.10199
0.2	0.38462	0.32625	0.27699	0.25537	0.23898	0.21966	0.20792	0.20398
0.3	0.55046	0.47841	0.41176	0.38119	0.35756	0.32926	0.31185	0.30596
0.4	0.68966	0.61803	0.54185	0.50458	0.47493	0.43854	0.41572	0.40792
0.5	0.80000	0.74150	0.66519	0.62433	0.59043	0.54731	0.51950	0.50986
0.6	0.88235	0.84482	0.77885	0.73856	0.70299	0.65525	0.62312	0.61176
0.7	0.93960	0.92266	0.87790	0.84384	0.81053	0.76170	0.72647	0.71358
0.8	0.97561	0.97031	0.95286	0.93261	0.90805	0.86492	0.82918	0.81522
0.9	0.99448	0.99321	0.99036	0.98698	0.98016	0.95799	0.92968	0.91622
1	1.00000	1.00000	1.00000	1.00000	1.00000	1.00000	1.00000	1.00000

**Fig. 3:**
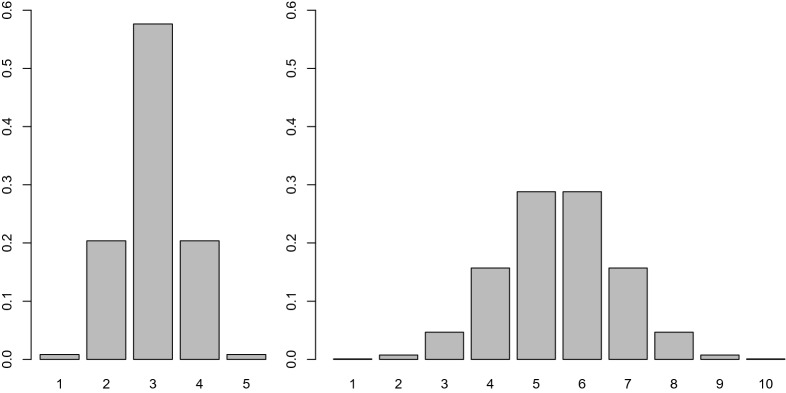
Barplot of two symmetrical non-uniform distributions, with 5 (left) and 10 (right) categories, obtained by mimicking the probability density function of a standard normal variable

We now move to non-uniform symmetrical ordinal distributions; in particular, we consider a probability mass function that somewhat resembles the probability density function of the normal variable. For this aim, the $(-4,+4)$ interval, whose probability for a standard normal variable is almost equal to 1, is divided into *H* equal-width adjacent intervals, $\left( -4+8(i-1)/H,-4+8i/H\right)$, $i=1,\dots ,H$. A similar construction was employed by Becker (1989) (who named it the “uniform cut-points (UCP) method” and employed it to study similarities between uniform association models and ordinalized bivariate normal distribution) and Ferrari and Barbiero (2012) (to study the effects of discretization of a bivariate normal distribution on the correlation coefficient). To the *i*-th interval, the *i*-th ordered category is associated, whose probability is thus equal to $\Phi (-4+8i/H)-\Phi (-4+8(i-1)/H)$, $i=1,\dots ,H$; the residual probabilities of $(-\infty ,-4)$ and $(4,+\infty )$ are assigned to the first and last categories, respectively. Thus, the probability distribution is always symmetrical about the central category (if *H* is odd) or categories (if *H* is even). The probabilities obtained according to this scheme are graphically displayed, for $K=5$ and $K=10$, in Fig. 3.

Table 4 reports the values of Goodman and Kruskal’s $\gamma$ for $\tau$ ranging from $-1$ to $+1$, with a step length of 0.1, and several values of the common number of categories of the two identical symmetrical margins. Note that as in the case of equiprobable categories, $\gamma$ is an odd function of $\tau$. For a given value of $\tau >0$ and for $K>3$, $\gamma$ is a decreasing function of *K*; for a given value of $\tau <0$ and for $K>3$, $\gamma$ is an increasing function of *K* (for $K=2$, we obtain again a uniform distribution with two equally probable categories). As *K* tends toward $\infty$, $\gamma$ converges to $\tau$, but much more slowly than in the case of uniform margins. When $K=100$, the relative difference between $\tau$ and $\gamma$ in is not negligible at all, attaining $4.5\%$. This fact can be easily explained by just looking at the barplot in the right side of Fig. 3: among the *K* categories, only the central ones have appreciable probabilities, so the “actual” number of categories is (much) smaller than *K*, and then a larger value of *K* is required to obtain the same difference between $\tau$ and $\gamma$ that is achieved in case of uniform distributions.

**Table 4:** Values of Goodman and Kruskal’s gamma for several combinations of $\tau$ and number of categories of the two identical marginal symmetrical distributions (uniform cut-points)

$\tau .K$	2	3	5	7	10	20	50	100
$-$1	$-$1	$-$1	$-$1	$-$1	$-$1	$-$1	$-$1	$-$1
$-$0.9	$-$0.99448	$-$0.99698	$-$0.99501	$-$0.99391	$-$0.99233	$-$0.98624	$-$0.9589	$-$0.935
$-$0.8	$-$0.97561	$-$0.9864	$-$0.97816	$-$0.97373	$-$0.96625	$-$0.92727	$-$0.86415	$-$0.83419
$-$0.7	$-$0.9396	$-$0.96561	$-$0.94663	$-$0.9343	$-$0.90815	$-$0.83339	$-$0.75986	$-$0.73083
$-$0.6	$-$0.88235	$-$0.93147	$-$0.89509	$-$0.86429	$-$0.81635	$-$0.72489	$-$0.65291	$-$0.62681
$-$0.5	$-$0.8	$-$0.8799	$-$0.81381	$-$0.76197	$-$0.70225	$-$0.60945	$-$0.54487	$-$0.52252
$-$0.4	$-$0.68966	$-$0.80262	$-$0.69807	$-$0.63458	$-$0.57406	$-$0.49032	$-$0.43628	$-$0.41811
$-$0.3	$-$0.55046	$-$0.6843	$-$0.55126	$-$0.4895	$-$0.43685	$-$0.36909	$-$0.3274	$-$0.31363
$-$0.2	$-$0.38462	$-$0.50907	$-$0.38064	$-$0.33245	$-$0.29397	$-$0.24663	$-$0.21834	$-$0.2091
$-$0.1	$-$0.19802	$-$0.27417	$-$0.19424	$-$0.16799	$-$0.14776	$-$0.12347	$-$0.10919	$-$0.10456
0	0	0	0	0	0	0	0	0
0.1	0.19802	0.27417	0.19424	0.16799	0.14776	0.12347	0.10919	0.10456
0.2	0.38462	0.50907	0.38064	0.33245	0.29397	0.24663	0.21834	0.2091
0.3	0.55046	0.6843	0.55126	0.4895	0.43685	0.36909	0.3274	0.31363
0.4	0.68966	0.80262	0.69807	0.63458	0.57406	0.49032	0.43628	0.41811
0.5	0.8	0.8799	0.81381	0.76197	0.70225	0.60945	0.54487	0.52252
0.6	0.88235	0.93147	0.89509	0.86429	0.81635	0.72489	0.65291	0.62681
0.7	0.9396	0.96561	0.94663	0.9343	0.90815	0.83339	0.75986	0.73083
0.8	0.97561	0.9864	0.97816	0.97373	0.96625	0.92727	0.86415	0.83419
0.9	0.99448	0.99698	0.99501	0.99391	0.99233	0.98624	0.9589	0.935
1	1	1	1	1	1	1	1	1

In the end, we consider strongly asymmetrical distributions, namely triangular distributions with *H* categories, where the probability of the *i*-th category is proportional to *i* (through some positive constant $\alpha$). Since under this assumption, $\sum _{i=1}^H p_i = \sum _{i=1}^H \alpha i=\alpha H(H+1)/2$, then $p_i=2i/[H(H+1)]$. Choosing this distribution as the marginal distribution of both *X* and *Y*, Table 5 reports the corresponding values of $\gamma$ for $\tau$ ranging from $-1$ to $+1$, with a step length of 0.1, and for several values of the common number of categories. One can notice that, differently from the two cases analyzed before, due to the asymmetrical nature of the margins, $\gamma$ is no longer an odd function of $\tau$, $\gamma (\tau )$ being generally different from $-\gamma (-\tau )$.

**Table 5:** Values of Goodman and Kruskal’s gamma for several combinations of $\tau$ and number of categories of the two identical marginal triangular distributions

$\tau .K$	2	3	5	7	10	20	50	100
$-$1	$-$1	$-$1	$-$1	$-$1	$-$1	$-$1	$-$1	$-$1
$-$0.9	$-$1	$-$0.99467	$-$0.99648	$-$0.99377	$-$0.98691	$-$0.96792	$-$0.93751	$-$0.92107
$-$0.8	$-$0.99933	$-$0.97729	$-$0.96629	$-$0.94876	$-$0.92616	$-$0.88070	$-$0.83783	$-$0.82001
$-$0.7	$-$0.98525	$-$0.94097	$-$0.90166	$-$0.86891	$-$0.83421	$-$0.77835	$-$0.73459	$-$0.71792
$-$0.6	$-$0.93982	$-$0.87473	$-$0.80945	$-$0.76715	$-$0.72757	$-$0.67084	$-$0.63032	$-$0.61554
$-$0.5	$-$0.85853	$-$0.77767	$-$0.69721	$-$0.65222	$-$0.61317	$-$0.56094	$-$0.52560	$-$0.51304
$-$0.4	$-$0.74150	$-$0.65396	$-$0.57108	$-$0.52900	$-$0.49425	$-$0.44974	$-$0.42065	$-$0.41048
$-$0.3	$-$0.59061	$-$0.50867	$-$0.43537	$-$0.40046	$-$0.37255	$-$0.33779	$-$0.31557	$-$0.30788
$-$0.2	$-$0.41063	$-$0.34734	$-$0.29328	$-$0.26853	$-$0.24914	$-$0.22540	$-$0.21041	$-$0.20526
$-$0.1	$-$0.20993	$-$0.17580	$-$0.14740	$-$0.13465	$-$0.12476	$-$0.11275	$-$0.10522	$-$0.10263
0	0	0	0	0	0	0	0	0
0.1	0.20632	0.17431	0.14685	0.13436	0.12462	0.11271	0.10521	0.10263
0.2	0.39722	0.34185	0.29120	0.26742	0.24857	0.22525	0.21039	0.20525
0.3	0.56388	0.49802	0.43112	0.39810	0.37130	0.33745	0.31551	0.30786
0.4	0.70149	0.63884	0.56453	0.52516	0.49212	0.44911	0.42053	0.41044
0.5	0.80905	0.76064	0.68892	0.64700	0.61008	0.55992	0.52540	0.51298
0.6	0.88837	0.85940	0.80082	0.76119	0.72370	0.66940	0.62999	0.61544
0.7	0.94297	0.93060	0.89462	0.86338	0.83011	0.77651	0.73411	0.71777
0.8	0.97707	0.97283	0.96064	0.94475	0.92278	0.87863	0.83717	0.81978
0.9	0.99482	0.99377	0.99154	0.98915	0.98460	0.96620	0.93662	0.92070
1	1	1	1	1	1	1	1	1

**Fig. 4:**
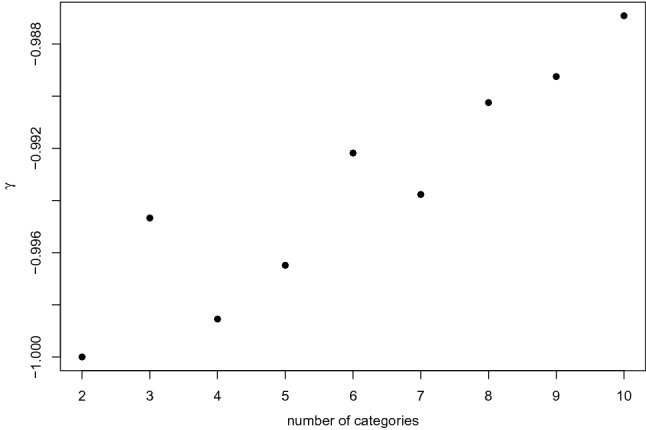
Kruskal’s $\gamma$ for an ordinalized bivariate normal random variable with Kendall’s rank correlation $\tau =-0.9$ as a function of the common number of categories (from 2 to 10) of the two ordinal triangular variables

For a given value of $\tau >0$, $\gamma$ is a decreasing function of *K*; for a given value of $-0.9<\tau <0$, $\gamma$ is an increasing function of *K*. When $\tau =-0.9$, we have a curious behavior of $\gamma$ as a function of *K*, which is displayed in Fig. 4.

It is worth underlining how the values of $|\gamma |$ are significantly larger than the corresponding $|\tau |$, especially when the number of categories is small. This feature is more apparent for negative values of $\tau$. For example, when $\tau =-0.5$ and $H=5$, the value of $\gamma$ is $-0.69721$. Even when discretizing the two continuous components through an extremely large number of ordered categories, such as 100, the relative difference between the values of $\tau$ and $\gamma$ is still non-negligible (not smaller than $2.6\%$ when $-0.9\le \tau \le 0.9$).

## Building a Bivariate Ordinal Variable with Prescribed Margins and Gamma Coefficient

Researchers can be interested in building and simulating samples from a bivariate (multivariate) ordinal distribution with prescribed marginal distributions and (pairwise) levels of association, expressed in terms of the gamma coefficient. Such a concern may arise when one has to show the appropriateness and study the performance or robustness of a novel statistical technique, since it is not always possible to do so by using analytic arguments, except in elementary cases; on the contrary, generating data replicates that mimic the real data’s characteristics of interest allows one to study the performance of the statistical method in any given setting. For example, researchers may be interested in assigning and preserving in their simulation study the marginal structures as well as ordinal associations (Demirtas 2006). In other circumstances, their concern is matching marginal means, variances, skewnesses and kurtoses, and intercorrelations (Vale and Maurelli 1983). van der Ark and van Aert (2015), with the aim of investigating the performance of different types of confidence intervals for $\gamma$, constructed several bivariate ordinal distributions by assigning each a fixed value of $\gamma$ and imposing constraints on the margins, considering uniform or skewed distributions.

However, when assigning the margins and association (correlation) value for two random variables, a uniqueness issue arises for the corresponding bivariate distribution. In fact, one of the fallacies of Pearson’s correlation is that if we consider a bivariate random vector (*X*, *Y*) with assigned marginal distributions $F_1$ and $F_2$ and a feasible value of $\rho$, then $F_1$, $F_2$, and $\rho$ do not in general determine the joint distribution *F* univocally; on the contrary, there may exist several (even infinite) joint distributions whose margins are $F_1$ and $F_2$ and whose linear correlation is $\rho$ (see, e.g., McNeil et al. 2005, p. 202). This issue is not overcome even by other dependence measures such as Spearman’s $\rho$ and Kendall’s $\tau$, nor most likely by Kruskal and Goodman’s $\gamma$. Nevertheless, if we restrict our focus to the family of joint distributions obtained by discretizing a specific class of continuous random vectors, we can find that there is a unique joint distribution satisfying the match of margins and association value.

Let us consider a bivariate ordinal variable with joint probabilities $p_{ij}$, $i=1,\dots ,H$, $j=1,\dots ,K$, and marginal probailities $p_{i\cdot }=\sum _{j=1}^K p_{ij}$ and $p_{\cdot j}=\sum _{i=1}^H p_{ij}$. Let us start with the simplest case: for a bivariate ordinal distribution with dichotomous margins, $H=K=2$, the problem consists of finding the solution to the following equation system:

$$\begin{aligned} {\left\{ \begin{array}{ll} p_{11}p_{22}(1-\gamma )&{}=p_{12}p_{21}(1+\gamma )\\ p_{12}&{}=1-p_{\cdot 1}-p_{22}\\ p_{21}&{}=1-p_{1\cdot }-p_{22}\\ p_{11}&{}=p_{1\cdot }-p_{12} \end{array}\right. } \end{aligned}$$

which becomes

$$\begin{aligned} {\left\{ \begin{array}{ll} (p_{1\cdot }+p_{\cdot 1}+p_{22}-1)p_{22}(1-\gamma ) &{}=(1-p_{\cdot 1}-p_{22})(1-p_{1\cdot }-p_{22})(1+\gamma )\\ p_{12}&{}=1-p_{\cdot 1}-p_{22}\\ p_{21}&{}=1-p_{1\cdot }-p_{22}\\ p_{11}&{}=p_{1\cdot }-p_{12} \end{array}\right. } \end{aligned}$$

and then

$$\begin{aligned} {\left\{ \begin{array}{ll} 2\gamma p_{22}^2 - p_{22} (1+3\gamma -2\gamma (p_{1\cdot }+p_{\cdot 1})) +(1+\gamma )\cdot (1-p_{1\cdot })(1-p_{\cdot 1}) &{}=0\\ p_{12}&{}=1-p_{\cdot 1}-p_{22}\\ p_{21}&{}=1-p_{1\cdot }-p_{22}\\ p_{11}&{}=p_{1\cdot }-p_{12} \end{array}\right. } \end{aligned}$$

From the first equation of the system above, we derive that $p_{22}$ is the unique real root (the one with minus sign) of the second-order equation $ax^2+bx+c=0$ with

$$\begin{aligned} {\left\{ \begin{array}{ll} a&{}=2\gamma \\ b&{}=-(1+3\gamma -2\gamma \cdot p_{1\cdot }-2\gamma \cdot p_{\cdot 1})\\ c&{}=(1+\gamma )\cdot (1-p_{1\cdot })\cdot (1-p_{\cdot 1}) \end{array}\right. }; \end{aligned}$$

the other joint probabilities can then be easily computed in cascade from the remaining equations. Thus, the problem has a unique feasible solution. For example, if we assign the margins $p_{1\cdot }=0.5$, $p_{\cdot 1}=0.5$, and set $\gamma =3/5$, we obtain the following second-degree equation: $3 x^2 - 4 x + 1$, whose roots are $p_{22,1}=1$ and $p_{22,2}=1/3$; taking the latter leads to the solution:

$$\begin{aligned} {\left\{ \begin{array}{ll} p_{22}&{}=1/3\\ p_{12}&{}=1/6\\ p_{11}&{}=1/3\\ p_{12}&{}=1/6 \end{array}\right. } \end{aligned}$$

Note that this set of probabilities corresponds to the joint distribution of the bivariate ordinal variable obtained by discretizing a bivariate normal (3) with $\rho =1/2$ (or, equivalently, $\tau =1/3$; see Eq. (4)).

For larger numbers of categories, there will be infinite solutions, which are not easy to derive, due to the nonlinear nature of the problem: the gamma coefficient is not linear in the $p_{ij}$’s, so the equation system is nonlinear, as we have already experienced with a $2\times 2$ contingency table. This means that there are several bivariate ordinal distributions $p_{ij}$ whose margins are the assigned $p_{i\cdot }$ and $p_{\cdot j}$ and whose gamma coefficient is equal to a prespecified value $\gamma \in [-1,+1]$. If one is interested in building just *one* bivariate ordinal variable with assigned margins and gamma, he/she can restrict his/her attention to the class of bivariate ordinal variables with the assigned margins derived by ordinalization of a standard bivariate normal distribution; then he/she can exploit the results of the previous section, by accommodating the value of the parameter $\tau$ in order to match the assigned value $\gamma$ between the assigned ordinal margins. Due to the monotonic relationship between $\gamma$ and $\tau$, whose form depends on the selected margins, and the fact that $\gamma$ can span its entire natural range $[-1,+1]$, which we proved empirically, we are confident that there will be a unique bivariate ordinalized distribution satisfying the requested requisites. This means that among all the bivariate distributions with assigned margins and $\gamma$, we select the unique one whose underlying continuous latent model is the bivariate normal.

Writing the relationship between the two association measures as $\gamma = g(\tau ;F_1,F_2)$, we just need to find the (unique) root $\tau$ of the equation $\gamma -g(\tau ;F_1,F_2)=0$, $\gamma$ being an assigned number in $[-1,+1]$ and $F_1$, $F_2$ being assigned ordinal distributions. Recovering the correct $\tau$ is a task that can be carried out by using an iterative procedure, which requires setting an initial value. Since we have empirically found that $\gamma$ after discretization is – in absolute value – larger than the rank correlation $\tau$ of the bivariate normal random variable, one can use $\tau ^{(0)}=\gamma$ as a starting value for the unknown $\tau$. One can then determine the corresponding value of the bivariate ordinal random variable $\gamma ^{(0)}$ and then iteratively adjust the value of $\tau$ according to some updating rule till $\gamma ^{(t)}$ converges to the assigned value. The updating process shall take into account that when $\tau$ is zero, $\gamma$ is zero, too, and should prevent the updated value of $\tau ^{(t)}$ from escaping the interval $[-1,+1]$; a proposal is suggested in the following algorithm:

1. Set $\tau ^{(0)}\leftarrow 0,\gamma ^{(0)}\leftarrow 0$; let $\epsilon >0$ be an arbitrarily small number
2. Set $$t\leftarrow 1$$ and $$\tau ^{(t)}\leftarrow \gamma $$
3. Compute $F(i,j;\tau ^{(t)})$ by using (6)
4. Compute $p(i,j;\tau ^{(t)})$ by using (7)
5. Compute $\gamma ^{(t)}$ for $p(i,j;\tau ^{(t)})$ by using (1)
6. If $|\gamma ^{(t)} - \gamma |<\epsilon$ stop; else
  set $$t\leftarrow t+1$$,
  $$\tau ^{(t)}\leftarrow \tau ^{(t-1)}+m^{(t)}(\gamma -\gamma ^{(t-1)})$$, with $$m^{(t)}=\dfrac{\tau ^{(t-1)}-\tau ^{(t-2)}}{\gamma ^{(t-1)}-\gamma ^{(t-2)}}$$;
  $$\tau ^{(t)}\leftarrow {\left\{ \begin{array}{ll} \min (\tau ^{(t)},1)&{} \text { if } \tau ^{(t)}\ge 0\\ \max (\tau ^{(t)},-1) &{} \text { if } \tau ^{(t)}< 0 \end{array}\right. };$$
  go back to 3.

Given the monotonic relationship between $\tau$ and $\gamma$ for the bivariate normal model and its ordinalized counterpart, the above algorithm should be able to recover the value of $\tau$ inducing the target $\gamma$ in a few steps, for any choice of $\gamma$ and of $F_1$ and $F_2$. The algorithm is implemented in the R environment and is freely available as supplementary material.

### Example 1

Consider the following margins for *X*: $p_{1\cdot }=p_{2\cdot }=p_{3\cdot }=p_{4\cdot }=0.25$, and for *Y*: $p_{\cdot 1}=0.1$, $p_{\cdot 2}=0.2$ $p_{\cdot 3}=0.3$, $p_{\cdot 4}=0.4$, and assign to $\gamma$ the value 0.5. After only five iterations, the algorithm illustrated above recovers the joint distribution ensuring the target $\gamma$ and the assigned margins, by ordinalization of a bivariate standard normal variable, which is displayed in Table 6.

**Table 6:** Joint distribution ensuring the target $\gamma =0.5$ and the assigned margins, by ordinalization of a bivariate standard normal variable

*X*, *Y*	1	2	3	4	total
1	0.0593	0.0782	0.0726	0.0399	0.25
2	0.0248	0.0600	0.0863	0.0789	0.25
3	0.0120	0.0415	0.0817	0.1147	0.25
4	0.0039	0.0203	0.0594	0.1664	0.25
Total	0.1	0.2	0.3	0.4	1

This joint distribution is not the unique one satisfying the requirements on the margins and gamma. A different one can be obtained as a convex combination of the cograduation (denoted by the letter “M”) and countergraduation (“W”) tables (Table 7):

**Table 7:** Cograduation (*M*, left) and countergraduation (*W*, right) tables based on the margins of the joint distribution displayed in Table 6

*X*, *Y*	1	2	3	4	Total	*X*, *Y*	1	2	3	4	Total
1	0.1	0.15	0	0	0.25	1	0	0	0	0.25	0.25
2	0	0.05	0.2	0	0.25	2	0	0	0.1	0.15	0.25
3	0	0	0.1	0.15	0.25	3	0	0.05	0.2	0	0.25
4	0	0	0	0.25	0.25	4	0.1	0.15	0	0	0.25
Total	0.1	0.2	0.3	0.4	1	Total	0.1	0.2	0.3	0.4	1

Note that being the distribution of *X* symmetrical, we have that $p^{M}_{i,j} = p^{W}_{I-i+1,j}$, $\forall i=1,\dots ,4$, with $I=4$

The joint probabilities $p_{ij}^* = \lambda p^M_{ij} + (1-\lambda ) p^W_{ij}$, with $\lambda =0.7293$, preserve the margins and ensure the target $\gamma$. It is worth underlining that the nonlinearity of $\gamma$ does not allow its direct derivation for a convex combination of two joint probability distributions. In fact, the value of $\gamma$ for a convex combination of two joint distributions is not equal to the same convex combination of the corresponding $\gamma$’s; in the case of combination of cograduation and countergraduation tables, it is not equal to $\lambda \cdot 1+(1-\lambda )\cdot (-1)=2\lambda -1$.

## Application to Inference

If a bivariate ordinal sample of size *n* is available, one can assume it is an *i.i.d.* sample from an ordinalized bivariate normal distribution, or, more generally, from an ordinalized bivariate continuous distribution whose unique copula is the Gaussian one (see, in this sense, Grønneberg and Foldnes 2019; Foldnes and Grønneberg 2019, for a more detailed account on identifiability issues). One has then to estimate the value of the unknown $\tau$ (or $\rho$) and those of the unknown thresholds that define the marginal distributions of *X* and *Y*; this can be done by numerically maximizing the joint log-likelihood of the observed sample with respect to all the unknown parameters simultaneously (see Olsson 1979, for details).

As an alternative to this full maximum likelihood approach, one can resort to the following method of moments for estimating the thresholds and $\tau$:

1. Compute the empirical cumulative distribution functions ${\hat{F}}_1$ and ${\hat{F}}_2$ based on the bivariate sample, and the thresholds by using the inverse cdf (see Sect. 3). Compute the sample value of $\gamma$, ${\hat{\gamma }}_M$, based on the bivariate sample.
2. Compute the value of $\tau$, ${\hat{\tau }}_M$, of the underlying bivariate normal distribution, inducing ${\hat{\gamma }}_M$ given ${\hat{F}}_1$ and ${\hat{F}}_2$, by resorting to the iterative procedure of Sect. 4. That is, since we can write $\gamma =g(\tau ;F_1,F_2)$ and $\tau =g^{-1}(\gamma ;F_1,F_2)$, ${\hat{\tau }}_M = g^{-1}({\hat{\gamma }}_M;{\hat{F}}_1,{\hat{F}}_2)$.

Given the way the estimates are derived, this method can be classified as a two-stage method of moments. The unknown parameters are in fact estimated in two sequential steps: first, the thresholds for $Z_1$ and $Z_2$ are estimated independently based on the empirical cdf of *X* and *Y*, respectively, and the sample value of $\gamma$ is calculated as well; then, the dependence parameter $\tau$ is estimated based on the quantities computed at the first stage.

A numerical example with artificial data is provided in the R code provided as supplementary material, where the two methods are implemented and compared.

## Real Data

In a now classic study of mental health in Manhattan, New York, Srole and Fischer (1978) explore the relationship, among others, between mental impairment (*Y*) and parents’ socioeconomic status (*X*). Table 8, from that study, has been used extensively to illustrate the utility and application of models for ordered categorical data.

**Table 8:** The Midtown Manhattan Study: Mental Health and Parents’ Socioeconomic Status

Parents’ Socioeconomic Status	Mental Health				
	Well	Mild symptom formation	Moderate symptom formation	Impaired	Total
A (high)	64	94	58	46	262
B	57	94	54	40	245
C	57	105	65	60	287
D	72	141	77	94	384
E	36	97	54	78	265
F (low)	21	71	54	71	217
Total	307	602	362	389	1660

The sample value of Goodman-Kruskal’s $\gamma$ is 0.15429. By assuming a bivariate standard normal distribution underlying the bivariate ordinal data, we can derive the maximum likelihood estimates (MLEs) for Kendall’s tau and the thresholds for the two margins (see Table 9). Note that the MLE of $\tau$ is 0.10762, quite a bit smaller than the corresponding estimate of $\gamma$, confirming the empirical results of the study in Sect. 3. However, the estimate is significantly greater than zero, with an associated standard error of 0.01718, which provides strong evidence that mental health status and parents’ socioeconomic status are positively associated (i.e., higher socioeconomic status is associated with better mental health), even if the strength of that association is quite small. Note also that all the MLEs of the thresholds are highly significant, except for $\theta _3$, which is the central threshold for the variable parents’ socioeconomic status.

**Table 9:** MLEs for the Midtown Manhattan Mental study data, assuming an ordinalized bivariate normal model

Parameter	Estimate	St. error
$\tau$	0.10762	0.01718***
$\theta _1$	$-$1.00344	0.03702***
$\theta _2$	$-$0.51024	0.03219***
$\theta _3$	$-$0.05579	0.03074
$\theta _4$	0.55185	0.03253***
$\theta _5$	1.12411	0.03900***
$\eta _1$	$-$0.89627	0.03573***
$\eta _2$	0.11931	0.03080***
$\eta _3$	0.72393	0.03386***

Signif. codes: 0 ’***’ 0.001 ’**’ 0.01 ’*’ 0.05 ’.’ 0.1 ’ ’ 1 (maximized value of the log-likelihood function: $-5171.345$). The results are obtained by using the package maxLik

If one applies the two-stage method of moments in Sect. 5, the estimate of $\tau$ is equal to 0.10665, just slightly different from the maximum likelihood estimate.

Table 10 displays the expected joint frequencies $n_{ij}^{(o)}$ under the bivariate ordinalized distribution whose parameters are set equal to the corresponding MLEs. Note that under this model, the marginal frequency distributions are not equal to the analogous ones of Table 8 (even if they are very close to each other). This is because the full MLEs of the thresholds are not equal to the corresponding marginal MLEs (which are used as starting values for the optimization routine). Comparing the observed and theoretical contingency tables, one can notice some slight discrepancies between homologous frequencies. In order to (approximately) evaluate the goodness-of-fit of the suggested ordinalized bivariate normal model to the data at issue, we computed the usual chi-squared statistic based on the theoretical and observed frequencies (all theoretical joint frequencies are greater than 5, so there is no need for pooling cells); its value is 8.84. Under the null hypothesis that the data come from the ordinalized bivariate normal distribution with parameters equal to their MLEs, this statistic approximately follows a chi-square distribution with a number of degrees of freedom equal to $24-9-1=14$, where 24 is the number of pooled frequencies and 9 is the number of estimated parameters. The corresponding *p*-value is 0.841, leading us to comfortably accept the null hypothesis. The value of the log-likelihood ratio statistic, given by $-2\sum _{i=1}^H\sum _{j=1}^Kn_{ij}\log (n_{ij}^{(o)}/n_{ij})$, is equal to 8.959 (*p*-value 0.834) and thus leads to the same conclusion.

**Table 10:** Expected joint frequencies under the ordinalized bivariate normal model whose parameters are obtained by applying the maximum likelihood method to the data of Table 8

*x*, *y*	Well	Mild	Moderate	Impaired	Total
A	67.8	102.0	50.1	42.1	262.0
B	53.0	93.0	50.7	47.5	244.2
C	55.8	106.8	61.9	62.3	286.9
D	65.8	138.8	86.2	93.9	384.7
E	39.1	91.8	61.5	73.3	265.7
F	25.6	69.3	51.5	70.2	216.6
Total	307.2	601.6	361.8	389.4	1660

Other more sophisticated models may fit the sample data better. For some examples of alternatives (namely, association models) fitting the Midtown Manhattan Mental study data, see for example Becker (2014). In particular, a uniform association model can be used: the expected cell frequencies can be calculated by using the vcdExtra R package (Friendly 2017) (see the supplementary material). Note that these expected frequencies are quite close to their analogs under the ordinalized bivariate normal model, confirming the findings of Becker (1989) and as stressed by Kateri (2014). To prove this, one can compute the Kullback-Leibler distances between the two theoretical joint distributions ($p_{ij}^{(o)}$ and $p_{ij}^{(u)}$, for the ordinalized normal and uniform association models, respectively): $K_1=\sum \sum p_{ij}^{(o)} \log (p_{ij}^{(o)}/p_{ij}^{(u)})$ and $K_2=\sum \sum p_{ij}^{(u)} \log (p_{ij}^{(u)})/p_{ij}^{(o)}$. The values are computed as $K_1=0.0001721$ and $K_2=0.0001724$, which are very small, proving that the two distributions are very close to each other.

## Conclusions and Further Research

We focused on the bivariate standard normal variable and studied the change in association before and after ordinalization, measured by Kendall’s $\tau$ and Goodman and Kruskal’s $\gamma$. This analysis has been facilitated (i) by the wide availability of software implementations of the bivariate normal cdf (despite its non-closed expression), which is required when computing the joint probabilities of the bivariate ordinal distribution resulting from discretization, and (ii) by the analytic relationship between Pearson’s correlation $\rho$ and Kendall’s $\tau$ for the bivariate normal distribution. We empirically investigated the relationship between $\tau$ and $\gamma$ by considering several specific configurations for the two final marginal distributions (uniform, unimodal or bimodal symmetric, triangular). The study confirmed a somewhat expected result, i.e., the association measure tends to inflate after discretization, and to a larger extent when the number of ordered categories is small. For a same number of categories, we also highlighted the effect of the marginal probabilities in the change of association. Based on these results, we also elaborated a scheme for building and simulating samples from a bivariate ordinal distribution with assigned margins and value of association.

Other bivariate continuous distributions or bivariate copulas can be explored when the interest is in analyzing the effects of ordinalization on Goodman and Kruskal’s $\gamma$ or in constructing a bivariate ordinal variable with assigned marginal distributions and association; one should prefer the parametric copula families satisfying conditions (i) and (ii) mentioned above. Moreover, one should focus on so-called “comprehensive” bivariate copulas (Nelsen 2006, p.15), i.e., copulas able to model continuously the whole range of dependence from the lower to the upper Fréchet bounds passing through the product copula. As for (i), there is a wide variety of parametric copulas with a closed-form expression of their joint cdf. As for (ii), an analytic expression linking the copula parameter to its Kendall’s correlation is not always available. For example, for the Frank family of copulas,

$$\begin{aligned} C(u_1,u_2;\theta )=-\frac{1}{\theta } \ln \left[ 1+\frac{(e^{-\theta u_1}-1)(e^{-\theta u_2}-1)}{e^{-\theta }-1}\right] ,\theta \in {\mathbb {R}}\setminus \left\{ 0 \right\} , \end{aligned}$$

we have the following relationship between Kendall’s $\tau$ and $\theta$: $\tau (\theta ) = 1 - 4[1-D_1(\theta )]/\theta$, with $D_1(x)=\frac{1}{x}\int _0^x \frac{t}{e^t-1}\text {d}t$, which can be computed numerically. Note that letting the parameter $\theta$ go to 0, the Frank copula boils down to the product copula, with $\tau (0)=0$; when $\theta \rightarrow -\infty$, *C* reduces to the countermonotonicity copula; when $\theta \rightarrow +\infty$, *C* reduces to the comonotonicity copula. The Plackett family of copulas has the following form:

$$\begin{aligned} C(u_1,u_2;\theta )=\frac{1+(\theta -1)(u_1+u_2)-\sqrt{[1+(\theta -1)(u_1+u_2)]^2-4\theta (\theta -1)u_1u_2}}{2(\theta -1)}, \end{aligned}$$

with $\theta \in (0,+\infty )\setminus \left\{ 1\right\}$. When $\theta \rightarrow 1$, it reduces to the product copula, whereas for $\theta \rightarrow 0$, it tends to the countermonotonicity copula, and for $\theta \rightarrow \infty$ to the comonotonicity copula. For this family, there does not appear to be a closed-form expression for Kendall’s $\tau$ (Nelsen 2006, p.171).

**Fig. 5:**
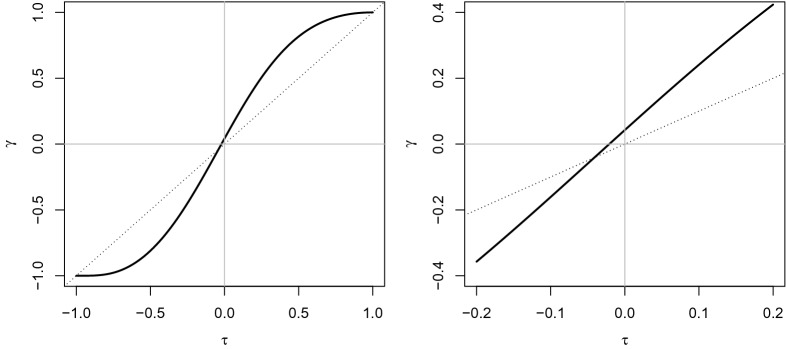
Relationship between $\tau$ and $\gamma$ for a bivariate Student’s *t* random variable before and after ordinalization (marginal probabilities for the two identical ordinalized variables are 1/3 and 2/3). In both graphs, the dotted line is the bisector of the first and third orthants. In the right graph, it can be better appreciated the behavior of $\gamma$ when $\tau$ is closer to zero

Another point to inspect is whether Kendall’s tau for a bivariate continuous distribution is always smaller – in absolute value – than Goodman and Kruskal’s gamma computed on any ordinalized version thereof. We have empirically shown that this happens for the bivariate normal distribution, in a certain sense reversing what happens with respect to the correlation coefficient, whose absolute value is always diminished by discretization of both components (Lancaster’s theorem). Actually, it happens that if we discretize the two components of a bivariate Student’s *t* distribution when $\rho =\tau =0$, the corresponding value of $\gamma$ between the two ordinalized variables is generally different from zero, so $\gamma$ does not generally inherit the sign of $\tau$, and the inequality $|\gamma |\ge |\tau |$ is no longer true. For example, if we consider two identical marginal distributions with probabilities 1/3 and 2/3 for the two ordered categories, then the bivariate Student’s *t* with 3 degrees of freedom and with uncorrelated components is discretized into the following bivariate ordinal variable:

$$\begin{aligned} \begin{array}{c|cc} X,Y &{} y_1 &{} y_2 \\ \hline x_1 &{} 0.1153 &{} 0.2180\\ x_2 &{} 0.2180 &{} 0.4487\\ \end{array} \end{aligned}$$

whose value of $\gamma$ is about 0.0424. In the graphs of Fig. 5, the relationship between $\tau$ and $\gamma$ is displayed for the ordinalized bivariate Student’s *t*. The reader can also refer to Jin and Yang-Wallentin (2017) for some potential alternatives to the bivariate normal distribution assumption as un underlying stochastic model for ordinal data, where the authors study robustness against misspecification of the underlying distribution with respect to the polychoric correlation estimation.

As an aside, the paper illustrated how bivariate ordinal models can be built for simulation studies, as an alternative to existing procedures employing categorical marginal models. Extension of the proposed procedures to the multivariate case is not straightforward. Analogous issues in determining the existence of a multivariate binary/ordinal variable with assigned margins and Pearson’s correlation matrix have been examined by Cario and Nelson (1997), Chaganty and Joe (2006), and Barbiero and Ferrari (2017). As we noticed, for a multivariate ordinal variable, the gamma association matrix is not necessarily positive semidefinite, so once all the margins are assigned, it is not straightforward to establish whether the assigned values of pairwise gamma coefficients lead to a feasible association matrix or not. However, if one selects a valid correlation matrix, this is a feasible Kendall’s tau correlation matrix for any choice of the (continuous) margins, and thus, per the arguments related to the change in magnitude after discretization, it should also be a feasible gamma association matrix for any choice of the ordinal margins. The points raised in this conclusive section can be addressed by future research.

## Supplementary material

Computer code developed in the R environment is available at https://tinyurl.com/PMET-D-19-00173.
